# Fit to learn: health and lifestyle predictors of academic performance in sports studies undergraduates

**DOI:** 10.3389/fpsyg.2026.1795078

**Published:** 2026-07-23

**Authors:** Mario Kasović, Bruno Škrinjarić, Tomáš Vespalec, Petra Vita Kasović, Ana Crnjak

**Affiliations:** 1 Faculty of Kinesiology, University of Zagreb, Zagreb, Croatia; 2 Faculty of Sports Studies, Masaryk University, Brno, Czechia; 3 The Institute of Economics Zagreb, Zagreb, Croatia; 4 School of Medicine, University of Zagreb, Zagreb, Croatia

**Keywords:** academic performance, energy drink consumption, physical fitness, sleep quality, students

## Abstract

**Introduction:**

Academic achievement is typically treated as a single outcome, obscuring the differing demands of theoretical and practical coursework. We examined how health, sleep quality, fitness, energy drink consumption, and sport status relate to performance across course types.

**Methods:**

Questionnaire data from 530 first-year kinesiology students (University of Zagreb, 2022/23–2024/25) were linked to official records of final grades in 11 courses. Ordinary least squares models with robust standard errors were estimated for overall GPA and six course-specific GPAs.

**Results:**

Males scored 0.21 points lower than females, most markedly in theoretical courses. Better self-rated health and fitness predicted higher grades. Sleep quality was unrelated to overall GPA but positively associated with team-sport grades. Energy drink intake showed a U-shaped association; professional athletes underperformed in theoretical courses.

**Discussion:**

Predictors of academic success operate differently across course types, supporting targeted academic support and health-promotion initiatives; the cross-sectional design precludes causal inference.

## 1 Introduction

Academic success in higher education must be viewed through a multidimensional lens, as it is not merely a reflection of student effort and intelligence, but rather a complex outcome of biological, socioeconomic, and contextual factors. While the biological foundation comprises health status, sleep quality, and energy drink consumption, which directly condition cognitive functions and stress levels (Okano et al., 2019), physical fitness and sport participation status dictate the physical capacities and time constraints uniquely faced by student-athletes (Stambulova and Wylleman, 2014). Additionally, material status dictates the level of financial pressure on the individual (Credé et al., 2010), while gender shapes specific learning styles and coping mechanisms. Crucially, these factors do not influence all academic demands equally; thus, performance must be analyzed across distinct course categories. Impaired sleep may drastically degrade performance on theoretical exams requiring abstract reasoning, whereas lower physical fitness will primarily jeopardize success in practical, motor-intensive courses (Hamlin et al., 2021; Solano et al., 2024).

Health and quality of life are not only subjective indicators of well-being (Burckhardt and Anderson, 2003; Jürges et al., 2008; World Health Organization, 1995) but also direct predictors of students’ cognitive capacity, motivation, and perseverance, which, in turn, shape their academic success. When a student rates their health as poor or experiences a low quality of life, it triggers a cascade of neurobiological, psychological, and behavioral barriers that directly impair academic performance, including grades and exam pass rates. Among sports science students, health and quality of life are directly linked to levels of physical activity and to the risk of overload. Balancing academic obligations and intense training schedules (dual career) can lead to overexertion and burnout syndrome, which dramatically undermines quality of life and academic engagement (Stambulova, 2017; Sorkkila et al., 2017). Low health is often associated with subclinical symptoms such as chronic fatigue, somatic complaints, and anxiety, which reduce cognitive resources available for learning and impair memory, attention, and decision-making (Kesin and İşsever, 2026). Quality of life also shapes academic outcomes by influencing intrinsic motivation and protecting against burnout. Poor living conditions, financial stress, or social isolation can increase cynicism, procrastination, and ultimately lower GPA (Torkani et al., 2025).

Sleep is a fundamental biological process that underpins physical recovery, emotional regulation, and cognitive functioning. Insufficient or poor-quality sleep is consistently associated with impaired attention, memory, and executive control, alongside adverse physical and mental health outcomes such as cardiometabolic risk, mood disturbances, and elevated stress, all of which can undermine academic engagement and performance (Scott et al., 2021). At the same time, lifestyle behaviors common among university students, particularly the consumption of energy drinks and other caffeinated stimulants, are closely intertwined with sleep disruption, anxiety, and stress, and show mixed and potentially non-linear associations with academic outcomes (Oglesby et al., 2018; Protano et al., 2023). Among university populations, the prevalence of suboptimal sleep is high and often peaks during assessment periods, when fatigue and sleep fragmentation rise and grades tend to fall (Bouloukaki et al., 2023). Studies report that better sleep quality, sufficient duration, and consistent timing are associated with higher academic performance (Okano et al., 2019), although some large-scale and longitudinal analyses reveal mixed or conditional effects, pointing to moderation by stress, mood, and study habits (Al Ani et al., 2024).

Physical fitness and participation in sports form a related but distinct pathway to academic outcomes. Among university students, higher fitness often correlates with mental well-being and cognitive performance (Chu et al., 2016). However, the relationship between elite athletic involvement and grades is complex: high-level athletes can benefit from discipline and time-management skills, but intensive training schedules may impose opportunity costs that, in theory, depress performance in theory-heavy courses (Comeaux and Harrison, 2011; Pinto-Escalona et al., 2022). Students enrolled in sports studies represent a distinctive population in this regard. On the one hand, regular physical activity and higher fitness levels are generally associated with enhanced cognitive functioning, which may support academic success (Donnelly et al., 2016; Chu et al., 2016). On the other hand, intensive training schedules, competitive sport commitments, and recovery demands can create trade-offs between athletic and academic goals. Prior research indicates that while physically active students often report better well-being, high-level or professional athletes may underperform in theory-heavy courses, highlighting the need to distinguish between different dimensions of academic performance rather than relying solely on overall grade point averages (Comeaux and Harrison, 2011; Pinto-Escalona et al., 2022).

Stimulant use, particularly energy drink consumption, is another widespread behavior among students. Cross-sectional and longitudinal studies have linked energy drink consumption to poorer sleep, higher anxiety, and mixed academic correlates. Some evidence suggests that energy drink consumers who study more also experience worse mental health outcomes, complicating causal interpretation (Oglesby et al., 2018; Oliveira Batista et al., 2025).

In terms of gender, female students in sports faculty achieve higher grades than male students, especially in theoretical domains (Kasović et al., 2021). Men may exhibit relative advantages in practical, performance-based assessments that align with greater sports competence (Turan et al., 2022), resulting in a pattern in which gender, sports skill, and assessment modality jointly influence academic outcomes.

Socioeconomic status (SES), encompassing material status and geographic origin (Operario et al., 2004), is a foundational external factor that decisively shapes students’ academic experiences and achievements (Reardon, 2011). Students from lower socioeconomic backgrounds face persistent existential pressure, as they are frequently compelled to work alongside their studies, drastically reducing the time available for learning, rest, and regeneration, thereby accelerating academic burnout (Brown et al., 2016). When this financial deficit intersects with geographic origin, particularly for students relocating to university centers from rural or economically disadvantaged areas, it introduces an additional, complex psychosocial burden. These students simultaneously contend with high housing costs and independent living, as well as the stress of intensive adaptation to a new, often alienating urban environment, which can induce feelings of isolation and ultimately result in a lower grade point average (Yigiter, 2025; Pyun and Malecki, 2026).

Importantly, academic achievement in sports studies is not a monolithic concept, as courses differ substantially in their cognitive, motor, and social requirements depending on their content and the nature of assessment (practical/team versus theoretical/mathematical). Theoretical courses place greater emphasis on declarative knowledge, abstract reasoning, complex problem-solving, and sustained concentration, whereas practical courses, especially those involving team sports, rely more heavily on motor learning, coordination, decision-making under time pressure, and interpersonal interaction (Evans et al., 2012).

Despite growing evidence linking health, sleep, physical fitness, stimulant use, and sociodemographic characteristics to academic success, relatively little research has examined whether these relationships vary systematically across course types within sports science programs. Existing studies typically treat academic achievement as a single outcome, most often measured by overall grade point average, thereby overlooking the distinct cognitive, motor, and social demands of theoretical and practical coursework. This limitation is particularly relevant in sports science, where students frequently balance academic responsibilities with intensive training and competitive sport commitments. Addressing this gap, the present study investigates how biological factors (sleep quality and physical fitness), lifestyle behaviors (energy drink consumption), and sociodemographic characteristics (gender, socioeconomic status, and place of origin) are associated with academic performance in theoretical and practical courses. By adopting a domain-specific perspective on academic achievement, this study contributes to the literature by identifying whether key predictors of success operate differently across course categories, thereby providing a more nuanced understanding of academic performance among sports science students.

## 2 Methodology

### 2.1 Data collection and sample

Data for this study come from two sources: (1) the survey on the sample of full-time first-year students of the Faculty of Kinesiology, University of Zagreb, Croatia, at the beginning of the second semester of the academic years between 2023 and 2025 (hereafter: SURVEY dataset); and (2) secondary data on academic performance of those students in the same academic years, obtained using the web-based modular information system (ISVU, 2025).

The SURVEY dataset includes 933 first-year students who participated in the study. Participants were selected based on enrollment in the first year of study at the Faculty of Kinesiology. Participation in the study was voluntary, and students were informed of the study’s purpose and methodology before completing the questionnaire. Survey data were collected via an online platform that provided easy access and anonymity, fostering trust and protecting participants’ privacy. Given an estimated cumulative enrollment of approximately 1,050 first-year students at the faculty during this period, this corresponds to an initial response rate of 88.9%.

The ISVU dataset contains records of academic performance during the first year of study for the 2022/2023, 2023/2024, and 2024/2025 academic years. Grades recorded in the ISVU database represent the final course grades awarded upon successful completion of a course, not the grades obtained on individual examinations. Final course grades are reported on a scale from 1 to 5, where 1 indicates a failed course and 2–5 represent passing grades, with 5 being the highest achievement. In Croatia, the academic year begins in the first week of October of the current year and ends in the first week of June of the following year. The academic year is divided into two semesters: the first (winter) semester runs from the start of the academic year to the end of January, while the second (summer) semester runs from the end of February to the end of the academic year.

Upon merging the SURVEY and ISVU datasets using unique, anonymous student matching codes, we are left with 530 students with complete academic records and survey data. Hence, the reduction from 933 to 530 observations was strictly due to administrative linkage constraints—namely, unmatched identification tokens, early university withdrawals, or incomplete examination marks at the time of data extraction.

Table 1 presents the sociodemographic and behavioral characteristics of the final sample of kinesiology students. The sample is predominantly male (63.6%), while females represent 36.4% of all respondents. Most students come from urban areas (61.9%), and their self-reported health and quality of life are generally high. Specifically, 85.8% of students report very good or excellent health, and 76.2% rate their overall quality of life as very good or excellent. In terms of material conditions, 85.5% of students assess their material status as below average or average, while 14.5% report it as above average. Regarding sports status, 57.4% of students identify as competitive athletes, 33.5% as recreational participants, and 9.1% as professionals. The average age of respondents is 19.5 years. The mean (reverse-coded) Pittsburgh Sleep Quality Index (PSQI) score equals 16.8 (the mean original PSQI score was 4.2), indicating generally good subjective sleep quality. For ease of interpretation, regression analyses use a reverse-coded version of the scale, where higher values indicate better sleep quality. On average, students consume 0.8 energy drinks per day and report an average fitness level of 6.9 on a 1–10 scale. Overall, the descriptive statistics indicate that the sample is relatively homogeneous in age and socio-economic background, predominantly male, physically active, and characterized by favorable health, lifestyle, and sleep patterns.

**Table 1:** Sample characteristics.

Respondent characteristics	*n*	%
Gender		
Female	193	36.4%
Male	337	63.6%
Residence status		
Rural (<10,000 residents)	202	38.1%
Urban (>10,000 residents)	328	61.9%
Health status		
Good	75	14.2%
Very good	297	56.0%
Excellent	158	29.8%
Material status		
Below average or average	453	85.5%
Above average	77	14.5%
Sport status		
Recreational	144	33.5%
Competitor	247	57.4%
Professional	39	9.1%

	Mean (Med.)	S. d.
Respondents’ age	19.5 (19)	0.8
Pittsburgh Sleep Quality Index (original)	4.2 (4)	1.9
Pittsburgh Sleep Quality Index (reverse-coded)	16.8 (17)	1.9
Respondents’ number of energy drinks daily	0.8 (1)	0.8
Respondents’ fitness	6.9 (7)	1.5

“S. d.” stands for standard deviation. “Med.” stands for median. Source: Authors’ work.

### 2.2 Empirical methodology and variables

Our empirical model is the following:

$$\begin{array}{l} {\mathrm{GPA}}_{i}=\alpha +\beta_{1}{\text{GENDER}}_{i}+\beta_{2}{\mathrm{AGE}}_{i}+\beta_{3}{\text{URBAN}}_{i}\\ +\beta_{4}{\text{HEALTH}}_{i}+\beta_{5}\mathrm{MAT}\_{\text{STATUS}}_{i}+\beta_{6}{\mathrm{LQ}}_{i}\\ +\beta_{7}{\text{PSQI}}_{i}+\beta_{8}{\mathrm{FIT}}_{i}+\beta_{8}{\mathrm{SPO}}_{i}+\beta_{10}{\mathrm{ENE}}_{i}+\beta_{11}{\mathrm{ENE}}_{i}^{2}+\varepsilon_{\mathrm{ic}} \end{array}$$ (1)

The dependent variable in our model is the students’ i grade point average (GPA). The covariates are divided into four subsets. The first set consists of students’ socio-economic characteristics: (1) gender (GENDER), (2) age (AGE), (3) type of settlement (URBAN), (4) health status (HEALTH), (5) material status (MAT_STATUS), and (6) general life quality (LQ). The second subset of variables contains a single variable that proxies for students’ sleep quality, measured using the Pittsburgh Sleep Quality Index (PSQI). The third subset captures students’ level of physical activity, measured using: (1) fitness level (FIT), and (2) an indicator of sport competitive status (SPO). The final subset of covariates controls students’ consumption of energy drinks in both linear (ENE) and quadratic forms. The model’s error term, $\varepsilon_{i}$ , is assumed to follow a normal distribution with mean zero and constant variance.

Our dependent variable, GPA, was calculated from students’ final course grades. The dataset comprises grades upon successful completion from 11 courses, split between the first and second semesters, enabling a detailed examination of students’ academic progress throughout the academic year (Table 2). Courses were categorized into two main groups: theoretical and practical (sports-related) courses. Practical courses were further subdivided into team and individual sports. This classification captures a broader spectrum of students’ academic development by combining their theoretical knowledge with practical sports competencies. Theoretical courses were further subdivided into general and mathematical/numerical courses. Sleep quality affects theoretical and overall grades differently: it is often more important for understanding and memorization, while motor coordination, reaction speed, and decision-making are particularly vital for practical assessments. Maintaining a healthy sleep pattern is crucial for optimal brain functioning and performance in both areas. Adopting healthy sleeping routines can help achieve better results in these areas.

**Table 2:** Courses in the first year.

No.	Semester	Course	Theoretical or practical course	Practical course subdivision	Theoretical course subdivision
1	Winter	Athletics – Walking and Running	Practical	Individual	
2	Winter	Functional Anatomy	Theoretical		General
3	Winter	Volleyball	Practical	Team	
4	Winter	Basic Kinesiological Transformations I	Practical	Individual	
5	Winter	History of Sport	Theoretical		General
6	Winter	Systematic Kinesiology	Theoretical		
7	Winter	Athletics – Throwing and Jumping	Practical	Individual	
8	Summer	Biomechanics	Theoretical		Mathematical
9	Summer	Quantitative Methods	Theoretical		Mathematical
10	Summer	Basic Kinesiological Transformations II	Practical	Individual	
11	Summer	Handball	Practical	Team	

Source: Authors’ work.

The Pittsburgh Sleep Quality Index (PSQI), a 19-question questionnaire, was used to assess sleep quality (Buysse et al., 1989). The scale for each component ranges from 0 to 3, with lower scores indicating the absence of problems and higher scores indicating worsening issues. By combining certain questions, seven main components are derived: subjective sleep quality, sleep latency, sleep duration, sleep efficiency, sleep disturbances, use of sleep medications, and daytime dysfunction. These components are summed to produce an overall score ranging from 0 to 21 points, with higher values indicating poorer sleep quality. To facilitate interpretation of regression coefficients, we reverse-coded the measure by subtracting the original PSQI score from 21, yielding a scale ranging from 0 to 21, with higher values indicating better sleep quality. Throughout the analysis, this reverse-coded PSQI measure is used.

The level of perceived physical fitness (FIT) was assessed using the following question: “How would you rate your physical fitness?” on a scale from 1 (very poor fitness) to 10 (excellent fitness). This question assesses participants’ overall physical readiness, including strength, endurance, flexibility, and agility. Fitness is crucial to participants’ ability to engage actively in various physical activities. Research has shown that this assessment is significantly associated with objectively measured physical fitness and perceived well-being (Plante et al., 2000; Gerber et al., 2013; Kasović et al., 2020).

Competitive sport status (SPO) specifies the level at which the student participates in sports, ranging from recreational activities for enjoyment (Recreational level), students who take sports seriously by participating in local leagues or competitions (Competitive level), and those who compete at the highest level as members of sports teams or clubs (Professional level). It is assumed that each level has distinct characteristics and poses different risks to students.

Energy drinks (ENE) represent the number of students who consume them per day. Respondents assessed their overall health (HEALTH) on a four-point scale ranging from poor to excellent. Previous research has shown that self-rated health is a reliable predictor of objective health outcomes and mortality (Jürges et al., 2008). Material status (MAT_STATUS) was operationalized as subjective socioeconomic status, measured through respondents’ assessment of their material conditions relative to the average population (below average, average, above average). This approach is particularly suitable for student populations, whose personal income often does not accurately reflect their socioeconomic position (Operario et al., 2004). Finally, life quality (LQ) captured respondents’ overall quality of life and subjective well-being using a four-point scale ranging from poor to excellent. This measure reflects the extent to which broader life circumstances, including financial conditions, housing, security, and social support, contribute to an individual’s overall life satisfaction (Phyo et al., 2020).

A description of all variables used in this model is presented in Table A1 in the Appendix.

The Ordinary Least Squares (OLS) method was employed to estimate the empirical model presented in Equation 1 because it is well-suited to analyzing continuous dependent variables, such as students’ GPA. OLS provides unbiased and efficient estimates under the classical linear regression assumptions, particularly when the residuals are homoscedastic and approximately normally distributed. Given the relatively large sample size and the continuous nature of both the dependent and explanatory variables, OLS is an appropriate, statistically robust approach for examining linear associations among sleep quality, socio-economic factors, physical activity, and academic performance. Moreover, the interpretability of OLS coefficients enables a clear assessment of the marginal effects of each covariate on academic outcomes, facilitating comparison with previous studies in educational and sports sciences, where OLS has been widely applied in similar contexts.

To verify the validity of the classical linear regression assumptions, several diagnostic tests were performed. We evaluated potential multicollinearity using Variance Inflation Factors (VIF). Because the model includes both a linear (ENE) and a quadratic term (ENE^2^) for energy drink consumption to capture non-linear relationships, structural multicollinearity is mechanically introduced between these two variables. To prevent this from distorting the diagnostic baseline, we calculated the VIFs using a model specification where the energy drink variable is mean-centered. Structural homoscedasticity of the error terms was formally evaluated using the Breusch-Pagan test. In instances where the null hypothesis of constant variance was rejected, the models were adjusted using Huber-White sandwich estimators to produce robust standard errors, guaranteeing the validity of our statistical inferences. Finally, while bounded GPA scales routinely yield non-normal test statistics in formal Shapiro–Wilk tests, the large sample size satisfies the asymptotic normality requirements of the Central Limit Theorem.

## 3 Results

Table 3 reports OLS estimates for seven alternative variations of dependent variables: GPA calculated over all first-year courses (column 1) and GPAs computed for subsets of courses (columns 2–7), specifically theoretical, practical, practical–team, practical–individual, theoretical–general, and theoretical–mathematical courses (as presented in Table 2). Each column shows estimated coefficients for the same set of covariates.

**Table 3:** Estimation results.

Regressors	All courses	Theoretical courses	Practical courses	Practical team courses	Practical individual courses	Theoretical general courses	Theoretical mathematical courses
	(1)	(2)	(3)	(4)	(5)	(6)	(7)
Gender (benchmark: female)							
Male	−0.210*** (0.046)	−0.316*** (0.078)	−0.164*** (0.047)	−0.245*** (0.073)	−0.102** (0.051)	−0.331*** (0.090)	−0.234** (0.098)
Age	0.037 (0.027)	0.074 (0.047)	0.019 (0.028)	−0.010 (0.042)	0.000 (0.032)	0.105* (0.056)	0.032 (0.055)
Settlement type (benchmark: rural)							
Urban	−0.010 (0.045)	0.022 (0.076)	−0.009 (0.045)	0.008 (0.068)	0.016 (0.048)	0.038 (0.090)	−0.052 (0.094)
Health status (benchmark: good)							
Very good	0.157** (0.065)	0.185 (0.113)	0.137** (0.062)	0.219** (0.094)	0.100 (0.065)	0.219 (0.135)	0.056 (0.135)
Excellent	0.055 (0.070)	0.048 (0.123)	0.074 (0.070)	0.042 (0.102)	0.088 (0.073)	0.083 (0.148)	−0.014 (0.141)
Material status (benchmark: below average or average)							
Above average	−0.097* (0.058)	−0.000 (0.100)	−0.145*** (0.054)	−0.217** (0.084)	−0.107 (0.066)	−0.044 (0.120)	0.186* (0.111)
PSQI (reverse-coded)	0.006 (0.011)	0.000 (0.018)	0.004 (0.011)	0.039** (0.016)	−0.013 (0.011)	−0.011 (0.021)	0.030 (0.021)
Fitness	0.046*** (0.017)	0.070*** (0.027)	0.038** (0.018)	0.005 (0.024)	0.056*** (0.019)	0.090*** (0.032)	−0.004 (0.036)
Sport status (benchmark: recreational)							
Competitor	0.039 (0.050)	−0.027 (0.082)	0.056 (0.051)	0.111 (0.074)	0.036 (0.054)	−0.078 (0.095)	0.165 (0.103)
Professional	−0.093 (0.082)	−0.416*** (0.137)	0.010 (0.082)	0.241** (0.121)	−0.108 (0.082)	−0.554*** (0.152)	0.058 (0.177)
Energy drinks	−0.271*** (0.072)	−0.416*** (0.116)	−0.189** (0.075)	−0.169 (0.109)	−0.154** (0.078)	−0.509*** (0.142)	−0.136 (0.149)
Energy drinks (squared)	0.082*** (0.027)	0.134*** (0.044)	0.057* (0.029)	0.042 (0.042)	0.042 (0.030)	0.170*** (0.058)	0.029 (0.062)
Constant	2.726*** (0.575)	1.742* (0.959)	3.277*** (0.597)	3.265*** (0.880)	3.889*** (0.690)	1.240 (1.153)	2.399** (1.066)
N	430	382	429	407	412	375	274
R squared	0.116	0.117	0.079	0.085	0.066	0.125	0.049

*p < 0.1, **p < 0.05, ***p < 0.01. Standard errors are shown in parentheses.

Several consistent patterns emerge. Male students have lower GPAs than females across virtually all specifications: the gender coefficient is negative and statistically significant in every model. In terms of interpretation, the point estimate in column 1 implies that, holding other covariates constant, male students score, on average, 0.21 grade points lower on the 2–5 GPA scale. This gap is larger in theoretical than in practical courses.

Self-reported health status is positively associated with academic performance: compared with the reference category “Good,” coefficients for Very good/Excellent health are positive and often significant, with particularly large estimates in the theoretical–general specification, while they do not show to be significant for practical courses.

Fitness is generally positively associated with GPA, with small but often significant coefficients (e.g., 0.046 in the all-courses model and 0.090 in theoretical general courses), indicating that higher self-rated fitness is associated with modestly better academic outcomes. Sport status (competitor/professional) is largely insignificant, except that professional status is associated with a lower GPA in theoretical and, especially, theoretical–general specifications (with estimated effects of −0.416 and −0.554, respectively), consistent with a potential trade-off between intensive sport commitments and theoretical coursework.

Sleep quality (measured by the reverse-coded PSQI, where higher values indicate higher sleep quality) shows a nuanced pattern: the overall GPA model (column 1) yields a near-zero, insignificant coefficient, while sleep quality is positively and significantly associated with performance in practical team courses (column 4, with an estimate of 0.039, meaning a one-point improvement in reverse-coded PSQI is associated with roughly 0.04 GPA points in team practical courses).

Energy-drink consumption exhibits a statistically significant non-linear association: the linear term is negative and highly significant across specifications, while the squared term is positive and significant (e.g., −0.271 and 0.082 across all courses, respectively). The implied turning point is approximately 1.6 drinks/day in the all-courses model, meaning that marginal effects are negative for consumption levels below ~1.6 drinks/day and become less negative (and, formally, positive beyond the turning point). Given the sample’s mean consumption (~0.8/day) and the limited support at high consumption levels, this non-linearity should be interpreted with caution.

There are also a few counterintuitive or unstable findings worth noting. The material status dummy is negative and significant in practical-course specifications (especially in practical team courses).

In summary, Table 3 highlights three insights for the present study: (1) a persistent male disadvantage in GPA that is strongest in theoretical subjects; (2) better self-reported health and higher fitness are generally linked to higher academic performance; and (3) sleep quality has a heterogeneous association depending on course type (positive and significant for team practical courses, no statistical significance for other courses), while energy-drink use shows a significant, non-linear relationship with GPA.

Regarding model diagnostics (Table A2 in the Appendix), VIFs were calculated for all model specifications. All VIFs were below 3.0, indicating that multicollinearity is not a concern. The Breusch–Pagan test revealed evidence of heteroscedasticity in several specifications (*p* < 0.05); therefore, all reported standard errors were estimated using Huber–White heteroscedasticity-robust estimators. Residual normality was assessed using the Shapiro–Wilk test. Although several specifications formally rejected the null hypothesis of normality, visual inspection indicated only minor departures from normality. Given the relatively large sample size and the use of heteroscedasticity-robust standard errors, inference is unlikely to be materially affected.

## 4 Discussion

This study examined how sleep quality, health, fitness, demographic factors, and stimulant use relate to the academic performance of first-year sports studies students across course types (theoretical vs. practical; team vs. individual). Three robust patterns emerged: (1) a persistent female advantage in GPA, particularly in theoretical courses; (2) consistently positive associations of self-reported health and perceived fitness with grades; and (3) a heterogeneous role for sleep quality and a complex, non-linear association between energy-drink consumption and academic outcomes.

Male students scored lower than females across most specifications (the largest gaps were observed in theoretical courses), which is in line with findings on gendered scholastic differences (Voyer and Voyer, 2014) and research showing that course type moderates gender gaps (Montolio and Taberner, 2015). Plausible mechanisms include differences in study organization, self-regulation, and sensitivity to assessment formats. The strong positive associations between better self-reported health and higher perceived fitness and GPA (especially in theoretical subjects) align with the literature linking physical health and aerobic fitness to executive function, attention, and memory (Donnelly et al., 2016). The observed positive associations may reflect several mechanisms discussed in the literature, including links between physical health, fitness, cognitive functioning, and class attendance. However, given the observational nature of the data, the present study cannot determine the direction of these relationships.

Contrary to previous studies reporting associations between poor sleep and lower academic performance (Okano et al., 2019), the PSQI showed no consistent association with overall GPA in our sample and was positively associated only with practical team courses. Several explanations are plausible. First, our sample displays relatively high and narrow PSQI scores (mean of 16.8, standard deviation of 1.9), which limits variability and statistical power to detect typical sleep–grade relationships. Second, the PSQI is a self-report measure that provides a one-month snapshot; transient exam-period changes or objective sleep metrics (such as actigraphy) may reveal different patterns (Fonseca and Genzel, 2020). Third, unmeasured mediators (depression, anxiety, stress) can confound or mediate sleep–performance links (Al Ani et al., 2024). The unexpected positive association for team practical courses may reflect selection effects, compensatory behaviors, or unobserved characteristics of students enrolled in these activities (e.g., students with demanding schedules organize teamwork differently); therefore, this finding should be interpreted with caution.

Energy drink consumption is associated with GPA in a U-shaped manner, with a negative linear term and a positive quadratic term indicating poorer outcomes at low-to-moderate intake and attenuation at very high intake. This pattern may reflect reverse causality (students struggling academically increase stimulant use), tolerance development, or selection effects rather than causal benefits of heavy consumption (Oglesby et al., 2018). Regardless, the known health risks of frequent energy drink use (such as cardiovascular risks, sleep disturbance, or anxiety) argue against interpreting the quadratic result as evidence of academic benefit. Our findings identify energy drink consumption as a significant correlate of academic performance; however, longitudinal studies are needed to establish temporal ordering and clarify the underlying mechanisms.

The quadratic U-shaped relationship between energy drink intake and GPA (Figure A1, Panel B) reveals a turning point at ~1.6 drinks daily. However, the sample distribution (Figure A1, Panel A) shows that most students cluster well below this threshold, with an average of 0.8 drinks. Thus, the upward tail lacks sufficient empirical support and should not be misconstrued as an academic benefit. Instead, this pattern reflects reverse causality: struggling, highly stressed students escalate stimulant intake as a compensatory mechanism to cope with heavy workloads. High consumption serves as a marker of student vulnerability rather than a true cognitive aid.

Professional athlete status was associated with lower GPAs in theoretical courses and higher GPAs in some practical/team courses. One possible explanation is that intensive training commitments create trade-offs with theoretical coursework, although the present data do not allow us to directly test this mechanism. This pattern is nevertheless consistent with findings from the dual-career literature (Comeaux and Harrison, 2011). The counterintuitive negative associations between above-average material status and practical course grades may reflect selection bias, resilience, or reporting bias. Students from lower socio-economic backgrounds who reach university can be highly motivated and may excel in applied/team contexts (Martí-Ballester, 2019). These nuances underscore the importance of multidimensional SES measurement (including parental education and income) and mixed-methods research to unpack the mechanisms.

## 5 Conclusion

This study investigated which individual health- and lifestyle-related factors (sleep quality, self-rated health and fitness, energy drink consumption, sports status, and sociodemographic characteristics) are associated with academic performance across different course types in first-year sports studies students. The objectives were to quantify these relationships, test for heterogeneity by course type, and draw implications for student support policy.

Three consistent patterns emerged. First, a persistent female advantage in GPA was evident across specifications (most pronounced in theoretical coursework). Second, better self-rated health and perceived fitness were robustly associated with higher grades, especially in theoretical subjects. Third, sleep quality showed weak, inconsistent associations with GPA in this sample, whereas energy drink consumption exhibited a nonlinear (U-shaped) relationship with academic outcomes. Professional athletes tended to underperform in theoretical courses but fared relatively better in some practical/team assessments.

Our findings suggest several practical steps for improving student success, targeting both broader factors, such as health and fitness, and more immediate ones, such as study habits and course scheduling. Firstly, universities should implement targeted academic support for male students in theory-heavy courses (e.g., study skills workshops). Additionally, universities should provide tailored support and flexible scheduling for student-athletes to mitigate conflicts between their academic and athletic careers. Our final policy recommendation is to implement pragmatic health education campaigns that highlight the limited academic benefits and known health risks associated with frequent energy drink use.

Ultimately, we must acknowledge the limitations of this research. Firstly, the degree of causal inference is limited by the study design. Sleep and stimulant use were measured cross-sectionally and by self-report, so temporal ordering and measurement precision are imperfect. Consequently, the observed relationships should be interpreted as associations rather than evidence of causal effects. Secondly, some covariate categories were small, reducing the precision of the estimates for those groups. Next, omitted-variable bias may be present, as important mediators and confounders (e.g., mental health, workload, class attendance) were not included and may account for some of the observed associations. Finally, our sample is from a single faculty, which may limit the generalizability of our findings.

## Data Availability

The datasets presented in this article are not readily available because authors do not have the permission to share used dataset. Requests to access the datasets should be directed to mario.kasovic@kif.unizg.hr.
