# Supplementary material for: Fit to learn: health and lifestyle predictors of academic performance in sports studies undergraduates
Source: Front Psychol. 2026 Jul 23;17:1795078. doi: 10.3389/fpsyg.2026.1795078 (PMC13463325; doi:10.3389/fpsyg.2026.1795078)
Supplement: Supplementary file 1 [file Supplementary_File_1.docx]

**Figure A1**. Distribution and predictive margins of energy drinks.

Notes: Shaded area on panel B represents 95% confidence interval.

Source: Authors’ work.
