# Supplementary material for: Fit to learn: health and lifestyle predictors of academic performance in sports studies undergraduates
Source: Front Psychol. 2026 Jul 23;17:1795078. doi: 10.3389/fpsyg.2026.1795078 (PMC13463325; doi:10.3389/fpsyg.2026.1795078)
Supplement: Supplementary file 2 [file Table_1.docx]

**Table A1**. Description of variables in the model.

| Variable | Description | Values |
| --- | --- | --- |
| *Dependent variable* |  |  |
| GPA | Students’ grade point average. | 1 (lowest) – 5 (highest) |
| *Independent variables* |  |  |
| Gender (GENDER) | Students’ gender. | 0 – Female, 1 – Male |
| Age (AGE) | Students’ age. | 18 – 23 |
| Type of settlement (URBAN) | The type of settlement the student comes from. | 0 – Rural, 1 – Urban |
| Health status (HEALTH) | Students' health status. | 1 (Bad) - … - 4 (Excellent) |
| Material status (MAT_STATUS) | Students' material status. | 1 – Below average, 2 – Average, 3 – Above average |
| Life quality (LQ) | Students' general life quality. | 1 (Bad) - … - 4 (Excellent) |
| Sleep quality (PSQI) | Students' sleep quality, proxied by the (reverse-coded) Pittsburgh Sleep Quality Index. | 1 (Worse) - … - 21 (Excellent) |
| Fitness (FIT) | Students’ fitness level. | 1 (Bad) - … - 10 (Excellent) |
| Sport status (SPO) | Students’ sports status. | 1 – Recreational, 2 – Competitor, 3 – Professional |
| Energy drinks (ENE) | The number of energy drinks students drink in a day. | 0 – 4 |

Source: Authors’ work.
