# Supplementary material for: Fit to learn: health and lifestyle predictors of academic performance in sports studies undergraduates
Source: Front Psychol. 2026 Jul 23;17:1795078. doi: 10.3389/fpsyg.2026.1795078 (PMC13463325; doi:10.3389/fpsyg.2026.1795078)
Supplement: Supplementary file 3 [file Table_2.docx]

**Table A2**. Diagnostic checks.

| Model | Testing for heteroscedasticity | | Testing for multicollinearity | | | Testing residuals | | |
| --- | --- | --- | --- | --- | --- | --- | --- | --- |
|  | Breusch-Pagan test | | Variance Inflation Factor | | | Shapiro–Wilk test | | |
|  | Chi2 value | p-value | | VIF mean | VIF max | | W value | p-value |
| All courses | 0.091 | 0.762 | | 1.363 | 2.482 | | 0.996 | 0.448 |
| Theoretical courses | 0.371 | 0.542 | | 1.365 | 2.472 | | 0.995 | 0.221 |
| Practical courses | 3.004 | 0.083 | | 1.362 | 2.474 | | 0.993 | 0.057 |
| Practical team courses | 8.992 | 0.003 | | 1.367 | 2.508 | | 0.980 | 0.000 |
| Practical individual courses | 0.147 | 0.701 | | 1.367 | 2.478 | | 0.989 | 0.004 |
| Theoretical general courses | 2.834 | 0.092 | | 1.365 | 2.511 | | 0.988 | 0.003 |
| Theoretical mathematical courses | 0.051 | 0.821 | | 1.343 | 2.271 | | 0.985 | 0.007 |

Source: Authors’ work.
